# Emergency department care experiences among members of equity-deserving groups: quantitative results from a cross-sectional mixed methods study

**DOI:** 10.1186/s12873-023-00792-z

**Published:** 2023-02-21

**Authors:** Susan A. Bartels, Meredith MacKenzie, Stuart L. Douglas, Amanda Collier, Jodie Pritchard, Eva Purkey, David Messenger, Melanie Walker

**Affiliations:** 1 Department of Emergency Medicine, Queen’s University, Kingston, ON Canada; 2 Department of Public Health Sciences, Queen’s University, Kingston, ON Canada; 3 Department of Family Medicine, Queen’s University, Kingston, ON Canada; 4 Street Health Centre, part of Kingston Community Health Centres, Kingston, ON Canada; 5 Department of Critical Care Medicine, Queen’s University, Kingston, ON Canada

**Keywords:** Addiction medicine, Disability, Emergency medicine, Equity-deserving groups, Health policy, Indigenous health, Mental health, Substance use disorder, Vulnerably housed, Visible minority

## Abstract

**Background:**

Emergency departments (EDs) serve an integral role in healthcare, particularly for vulnerable populations. However, marginalized groups often report negative ED experiences, including stigmatizing attitudes and behaviours. We engaged with historically marginalized patients to better understand their ED care experiences.

**Method:**

Participants were invited to complete an anonymous mixed-methods survey about a previous ED experience. We analysed quantitative data including controls and equity-deserving groups (EDGs) - those who self-identified as: (a) Indigenous; (b) having a disability; (c) experiencing mental health issues; (d) a person who uses substances; (e) a sexual and gender minority; (f) a visible minority; (g) experiencing violence; and/or (h) facing homelessness - to identify differences in their perspectives. Differences between EDGs and controls were calculated with chi squared tests, geometric means with confidence ellipses, and the Kruskal-Wallis H test.

**Results:**

We collected a total of 2114 surveys from 1973 unique participants, 949 controls and 994 who identified as equity-deserving. Members of EDGs were more likely to attribute negative feelings to their ED experience (p < 0.001), to indicate that their identity impacted the care received (p < 0.001), and that they felt disrespected and/or judged while in the ED (p < 0.001). Members of EDGs were also more likely to indicate that they had little control over healthcare decisions (p < 0.001) and that it was more important to be treated with kindness/respect than to receive the best possible care (p < 0.001).

**Conclusion:**

Members of EDGs were more likely to report negative ED care experiences. Equity-deserving individuals felt judged and disrespected by ED staff and felt disempowered to make decisions about their care. Next steps will include contextualizing findings using participants’ qualitative data and identifying how to improve ED care experiences among EDGs to make it more inclusive and better able to meet their healthcare needs.

**Supplementary Information:**

The online version contains supplementary material available at 10.1186/s12873-023-00792-z.

## Introduction

Emergency departments (EDs) can play an integral role in healthcare provision for patients who identify as being members of equity-deserving groups (EDGs). EDGs are defined as those who face significant collective challenges in participating in society due to attitudinal, historical, social, and environmental barriers based on age, ethnicity, disability, economic status, gender, race, and/or sexual orientation, among others [1]. They often identify barriers to equal access, opportunities, and resources due to discrimination. The importance of EDs in providing critical medical care to members of EDGs is particularly relevant in settings where access to primary care is limited, and the ED becomes a safety net. In fact, poverty, homelessness, social isolation, and marginalization have all been shown to be associated with increased ED use [2–5].

Some groups of marginalized patients have reported concerns about care received in the ED, including perceptions that they felt judged, their medical issues were not taken seriously, communication was disrespectful, and they were subjected to stigmatizing attitudes [5–10]. Negative experiences such as these can result in care avoidance and worsening health issues, which can be particularly detrimental for vulnerable patients [6–8]. Recent incidents involving the maltreatment of members of EDGs in Canadian EDs have drawn media attention and highlighted systemic racism within the health care system. For instance, Joyce Echaquan, a 37-year-old Indigenous woman from the Atikamekw Nation videoed and live streamed her treatment in a Quebec ED in 2020. In the video, staff are heard voicing racist slurs against Echaquan before she died under their care [9]. In British Columbia, healthcare workers were alleged to have assumed that Indigenous patients were intoxicated resulting in delayed and/or substandard care [10]. Additionally, several patients have recently spoken out about racism experienced in Canadian EDs, including differential treatment, delays in care, and assumptions about drug-seeking [11, 12].

While anecdotal evidence exists, empirical data to document and understand systemic racism within Canadian healthcare is lacking. Existing research primarily consists of smaller qualitative studies, often without the inclusion of controls to provide insights about how EDGs perceive their care in comparison to those who do not identify as equity-deserving [6, 7, 13, 14]. The Kingston Health Sciences Centre’s (KHSC) ED and urgent care centre (UCC) serve as the sole points of emergency care provision for patients who are members of EDGs in Kingston, Ontario, a city recognized for its income and quality of living disparities [15]. Yet, little is known about the experiences and perceptions of ED care among EDGs in this setting. We therefore engaged with historically marginalized patients to better understand their ED care experiences. Here we present the quantitative findings from this research with the qualitative results for each EDG to be published separately.

## Methods

### Study design

A participatory, mixed methods, cross-sectional study using sensemaking methodology with Spryng.io. Spryng.io is a narrative capture tool based on the premise that storytelling is a natural way to convey complex information and is used by individuals to make sense of their own and their community’s experiences [16–19]. Sensemaking is based on complexity theory and the Cynefin framework [20].

### Survey

Using Spryng.io, participants audio-recorded a brief narrative in response to an open-ended prompting question asking about a *past* ED /UCC experience occurring *within the previous 24 months*, thus generating the qualitative data. After the recording, participants then interpreted the shared experiences through a series of questions that asked them to plot their perspectives between three variables (triads) or using sliders (see Appendix 1 for examples). Spryng.io then quantifies each of the plotted points, providing statistical data along with the accompanying explanatory narratives [21]. Multiple-choice questions collected sociodemographic information, allowing patients to identify as members of EDGs who face barriers to accessing care in addition to experiencing stigmatizing attitudes and behaviours (Indigenous, visible minorities, persons with a disability, mental health problems, substance use disorder, vulnerably housed, experiencing violence, and sexual and gender minorities). Additional multiple-choice questions helped to contextualize the shared story. The list of EDGs is not exhaustive but these groups were identified *a priori* as the focus for this research. Those who experience interpersonal violence were included because they often feel ashamed, and risk being blamed for what happened to them. By collecting many self-interpreted stories, sensemaking methodology leverages the “wisdom of the crowds,“ and collectively, the participants’ interpretation responses create a nuanced picture in the same way pixels come together to produce a clear image [22]. Each survey took approximately 15 min to complete. The survey was developed by the research team in close collaboration with community partners (Appendix 2) and with input from individuals who identified with each of the EDGs. The full survey is provided in Appendix 3. Participants could share and interpret as many previous ED experiences as they wanted as long as the ED visits were within 24 months. With each individual survey submission, participants were asked to indicate the number of times they had participated (1, 2, 3, 4, etc.). The number of first submissions was taken to be the total unique number of participants.

### Setting

Data were collected at a single urban ED and a single urban UCC in Kingston, Ontario, Canada from June to August 2021, with annual patient volumes of 57,648 and 37,708, respectively. Trained research assistants (RAs) collected data at both sites from 9am to 9pm Monday to Friday. Patients who were medically unstable, who were aggressive or threatening towards staff, or who did not have the capacity to give informed consent were not approached. To include participants who were not actively seeking care in the ED/UCC, potentially due to prior negative experiences, RAs also collected data from clients visiting community partners including the Kingston Street Health Centre, Home Based Housing, St. Vincent de Paul, the Kingston Youth Shelter, and the Integrated Care Hub, among others. All data were collected in English using the Spryng.io app on handheld tablets. Surveys were completed either in the ED, UCC, or at a community partner’s office and there was no follow up or additional contact made with participants.

### Participants

Over a 3-month period, any medically stable patient, aged 16 and older, with adequate English fluency registering in the ED or UCC during study hours was invited to complete the survey regardless of self-identification as a member of an EDG. Survey completion was facilitated by an RA when necessary. Participants were able to self-identify as belonging to up to three EDGs that were most relevant to the ED care experience shared. Please see Appendix 2 for a complete list of EDGs named in the survey. Individuals who lacked capacity to give informed consent were not enrolled. For potential participants who were approached but declined participation, the reason for declining was recorded.

### Primary measures/outcomes

The primary outcome was self-described experiences in the ED comparing those who identified as being a member of at least one EDG to those who did not.

### Analysis

Descriptive statistics were calculated in SPSS (IBM SPSS Statistics V.26.0.0.0) with chi squared tests to identify differences in participant characteristics between EDGs and controls. P-values < 0.05 were taken to be statistically significant and do not include missing data. Prefer not to say/do not know responses were recoded as missing for the purpose of descriptive statistics. Sensemaking data were exported to Tableau (V.2020.4) where collective plots were visually analysed to identify patterns such as clusters of responses [16]. Triad and slider data were disaggregated based on whether a participant self-identified as being a member of an EDG or not. Triad data were further disaggregated based on which EDGs participants identified with. Geometric means for controls and each EDG subgroup were produced in R Scripts (R V.3.4.0) We also used R Scripts to generate 95% confidence intervals, which are presented graphically as 95% confidence ellipses [23, 24]. We deemed two geometric means to be statistically different if their 95% confidence ellipses did not overlap. Slider data was generated graphically as histograms and the collective areas under the bars for each subgroup was analyzed in SPSS (IBM SPSS Statistics V.26.0.0.0) using the Kruskal-Wallis H test and chi-squared tests to determine if the bar areas were statistically different between groups [25, 26]. Distributions of responses for the slider questions are presented as violin plots, with an asterisk indicating the overall mean for each sub-group.

### Ethical considerations

The study and survey were designed in collaboration with members of EDGs and community-based organizations including Indigenous partners led by ownership, control, access, and possession (OCAP) principles [27]. Informed consent was obtained from all participants by asking the participant to tap a box on the tablet. No identifying information was collected, and data were anonymous from the point of collection. Participants were offered a $5 coffee gift card as a token of appreciation. The study protocol was approved by the Queen’s University Health Sciences and Affiliated Teaching Hospitals Research Ethics Board (#6,029,400).

## Results

### Characteristics of study participants

A total of 4414 potential participants were approached, with 2579 (58.4%) declining to take part in the study, giving a response rate of 41.6%. Overall, 47.1% of participants completed the survey in the ED, 43.9% completed it in the UCC, and 9% completed it through a community partner independent of the ED and UCC. Of those who declined to participate, 1216 (46.9%) had not had a previous visit to the ED or UCC within the past 24 months, 488 (18.8%) were simply not interested, 380 (14.7%) felt too unwell to take part, and 169 (6.5%) had already participated.

Overall, 1973 unique participants completed the survey sharing a total of 2114 experiences about their ED care, including 949 participants who did not identify as equity-deserving and 994 who did identify as equity-deserving. Demographic details disaggregated by equity-deserving status are provided in Table 1. In total, 1740 participants (82.3%) shared a first person experience in the ED and another 154 (7.3%) shared their child’s experience in the ED. Members of EDGs were more likely to struggle to meet their needs (i.e., food, housing, clothing) (p < 0.001), attribute negative feelings to their ED experience (p < 0.001), indicate that their identity impacted the care received (p < 0.001), and say that they felt disrespected and/or judged while in the ED (p < 0.001).

**Table 1:** Study population characteristics disaggregated by self-identification as equity-deserving or not

	Total Interviews (% of N = 2114)	Identifies as Equity-Deserving (% of N = 994)	Does Not Identify as Equity-Deserving (% of N = 949)	p-value^a^
Gender Identity				
Female	1147 (54)	536 (54)	549 (58)	**< 0.01**
Male	840 (40)	394 (40)	375 (40)	
Non-binary	29 (1)	21 (2)	5 (1)	
Not sure / prefer not to say	98 (5)	43 (4)	20 (2)	
Total	2114	994	949	
Age				
< 18	137 (6)	33 (3)	93 (10)	**< 0.0001**
18–25	253 (12)	131 (13)	93 (10)	
26–45	455 (22)	251 (25)	167 (18)	
46–65	379 (18)	201 (20)	166 (17)	
> 65	266 (13)	108 (11)	153 (16)	
Missing data	624 (30)	260 (26.2)	267 (28.1)	
Total	2114	994	949	
Ethnicity				
White/European	1165 (55)	510 (51)	590 (62)	**< 0.0001**
Indigenous	99 (5)	87 (9)	9 (1)	
One or more ethnicity	40 (2)	31 (3)	7 (1)	
Black	26 (1)	21 (2)	4 (0)	
Other	114 (5)	59 (6)	39 (4)	
Missing data	670 (32)	286 (29)	300 (32)	
Total	2114	994	949	
Equity-deserving Group				
Indigenous	129 (7)	129 (13)	-	-
Having a disability	370 (19)	370 (37)	-	
Mental health issue	428 (22)	428 (43)	-	
Illicit substance use	246 (13)	246 (25)	-	
LGBTQI2S+	118 (6)	118 (12)	-	
Facing homelessness	171 (9)	171 (17)	-	
Visible minority	117 (6)	117 (12)	-	
Experience of violence	65 (3)	65 (7)	-	
History of incarceration	39 (2)	39 (4)	-	
Member of a gang	15 (1)	15 (2)	-	
Trading sex for money/goods	12 (1)	12 (1)	-	
Other	43 (2)	43 (4)	-	
No EDG	949			
Not sure / prefer not to say	171 (8)	0	-	
Total	2873	994	-	
Frequency With Which Patient Struggles to Make Ends Meet				
Never	861 (41)	294 (30)	524 (55)	**< 0.001**
Rarely	328 (16)	146 (15)	155 (16)	
Sometimes	332 (16)	174 (18)	131 (14)	
Often	162 (8)	114 (11)	34 (4)	
All the time	227 (11)	189 (19)	32 (3)	
Not sure / prefer not to say	204 (10)	77 (8)	73 (8)	
Total	2114	994	949	
Number of Visits to the ED in Preceding 24 months				
Did not visit	343 (16)	145 (15)	186 (20)	**< 0.001**
1–3 times	700 (33)	309 (31)	335 (35)	
≥ 4 times	244 (12)	170 (17)	63 (7)	
Missing data	827 (39)	370 (37)	365 (38)	
Total	2114	994	949	
How Personal Situation, Identity, and Culture Impacted the Care Experience				
In a very bad way	108 (5)	103 (10)	4 (0)	**< 0.001**
In a bad way	162 (8)	141 (14)	18 (2)	
It did not impact care	1329 (63)	552 (56)	715 (75)	
In a good way	132 (6)	67 (7)	50 (5)	
In a very good way	72 (3)	32 (3)	29 (3)	
Not sure / prefer not to say	311 (15)	99 (10)	133 (14)	
Total	2114	994	949	
Feelings About the ED Experience				
**Positive	1003 (47)	393 (40)	550 (58)	**< 0.001**
***Negative	706 (33)	410 (41)	242 (26)	
Mixed positive / negative	222 (11)	129 (13)	79 (8)	
Missing data	184 (9)	62 (6)	78 (8)	
Total	2114	994	949	
Shared Experience was About Lack of Respect and/or Judgement				
No	1487 (70)	615 (62)	787 (83)	**< 0.001**
Yes	396 (19)	291 (29)	85 (9)	
Not sure / prefer not to say	231 (11)	88 (9)	77 (8)	
Total	2114	994	949	

Differences between EDGs and controls were calculated with chi squared tests

P-values do not include missing data. Prefer not to say/do not know responses were recoded as missing for the purpose of descriptive statistics

*171 surveys did not indicate whether the patient identified as being a member of an equity-deserving group or not and were therefore omitted from the statistical analysis

** Positive emotions included feeling accepted, happy, hopeful, relieved, satisfied, or thankful

*** Negative emotions included feeling afraid, disappointed, embarrassed, frustrated, helpless, or worried

^ Participants could indicate that they self-identified as being members of up to 3 equity-deserving groups and therefore sums to greater than total number of individuals identifying as equity-deserving

### Summary of findings from triad questions

**Fig. 1:**
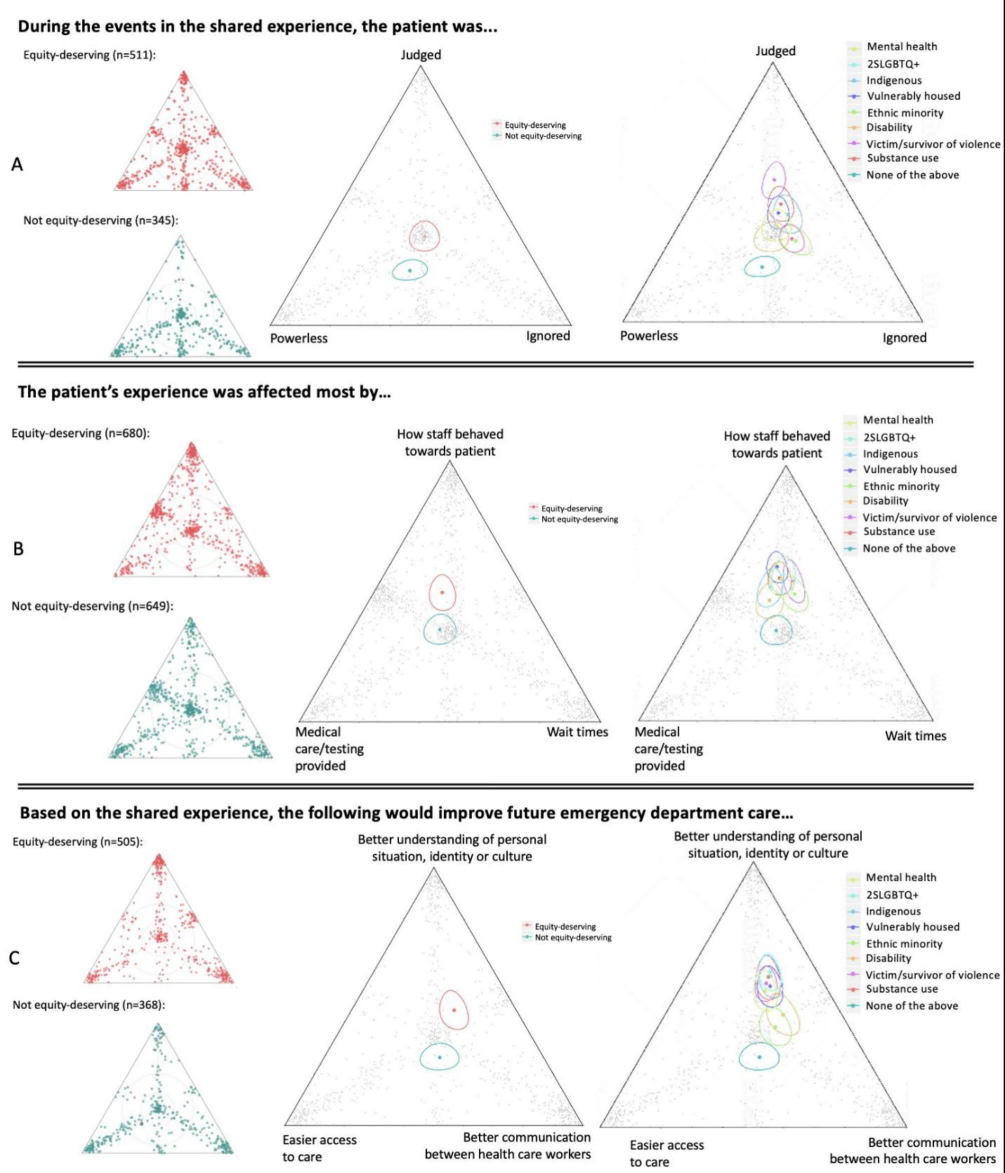
Geometric means with surrounding 95% confidence ellipses are provided on the right. Responses were statistically different between equity-deserving and non-equity-deserving groups as demonstrated by the non-overlapping 95% confidence ellipses, with participants who identified as equity-deserving more likely to indicate that they felt judged in the ED.

Participants who identified as equity-deserving were statistically more likely to report that they felt judged while receiving care in the ED (Fig. 1A), that their ED experience was affected by staff behaviour (Fig. 1B) and that better understanding of their personal situation, identity and culture was needed to improve care (Fig. 1C). Of note, participants did not perceive that the ED experience was significantly impacted by the medical care/testing received or by wait times. These patterns were true across all EDGs except for persons with disabilities who were not statistically more likely than controls to indicate that their experience was affected by staff behaviour (Fig. 1B).

### Summary of findings from slider questions

**Fig. 2:**
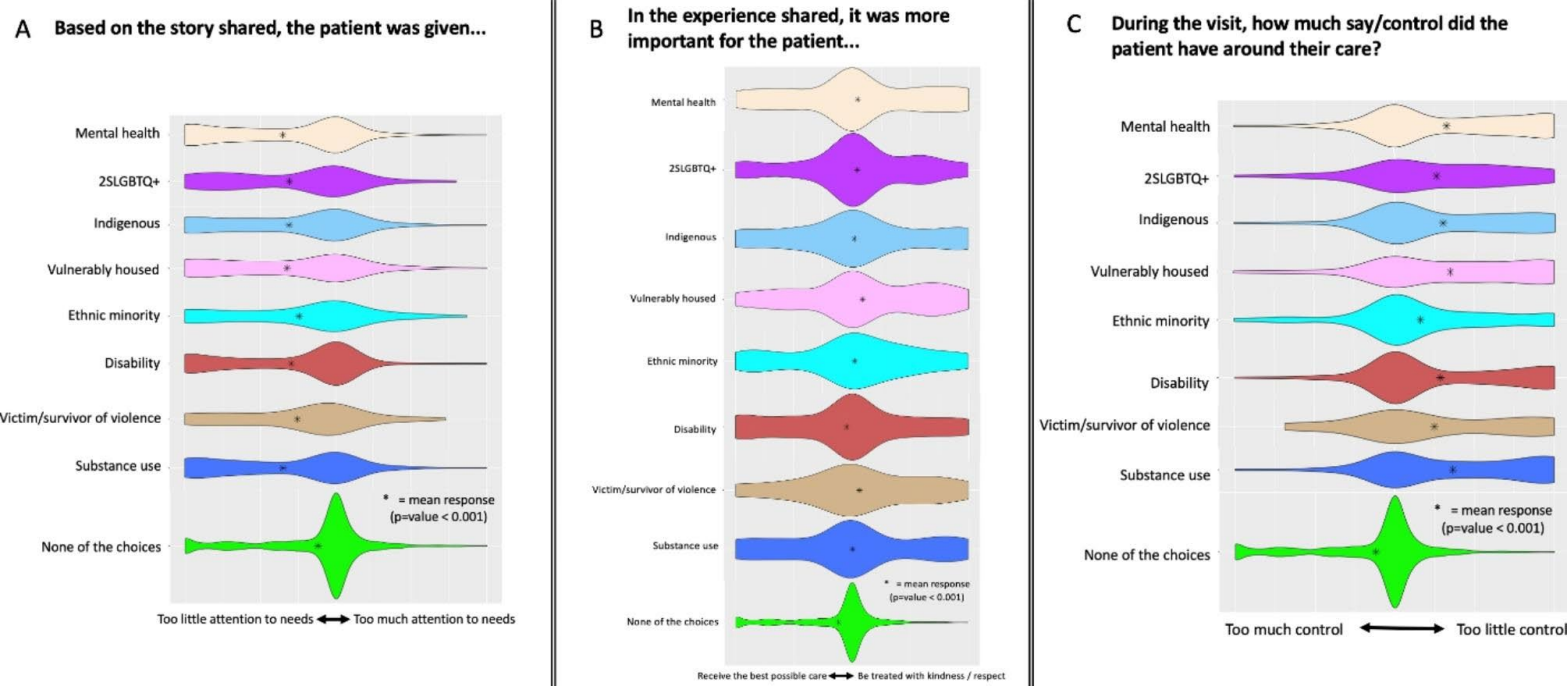
Asterisks indicate the overall mean for each sub-group and highlight that patients who identify as equity-deserving were more likely to indicate that their needs received too little attention (p < 0.001 for all 3 panels)

Members of EDGs were more likely to indicate that staff paid too little attention to their needs (p < 0.001) (Fig. 2A), that it was more important to be treated with kindness/respect than to receive the best possible care (p < 0.001) (Fig. 2B), and that they had little control in making decisions about their care (p < 0.001) (Fig. 2C).

## Discussion

Study findings suggest that members of EDGs were more likely to report negative ED care experiences than controls. Quantitative results highlight that equity-deserving individuals felt judged and disrespected by ED staff, believed that there was insufficient attention to their needs, and felt disempowered to make decisions about their care. We identified two areas that can be most easily addressed to improve care for members of EGDs: (1) improved recognition and attention to patients’ identity, culture, and personal situation and how that impacts care; and (2) treating all patients with kindness and respect.

Earlier research found that members of EDGs, such as Indigenous peoples [28, 29] and the vulnerably housed [6, 14], perceived that they experience longer wait times in the ED. However, our study does not support these findings, with members of EDGs not perceiving that they had different wait times than controls (Fig. 1B). It is noteworthy though that our findings are consistent with those of Batta et al. who found that wait times for Indigenous and non-Indigenous patients were similar in a Saskatoon ED among patients presenting with abdominal pain and triaged as a CTAS 3 [30]. It is also noteworthy that our study did not identify perceived differences between EDGs and controls with respect to diagnostic testing or medical care received (Fig. 1C). This contradicts some earlier published case reports [9, 31], and will also be examined in more detail. In the current analysis, the totality of observed differences was related to behaviors/attitudes rather than overtly inequitable medical care and services. ED perceptions of stigma and feeling judged have been commonly reported among sexual and gender minorities [32, 33], those who are vulnerably housed [6, 14, 34], Indigenous patients [28, 35, 36], patients with mental health concerns [37–39] and patients with substance use disorder [34]. Consistent with existing literature, the current findings also highlight that members of EDGs felt judged and disrespected while accessing ED care.

We believe that training and raising awareness for health care providers and working to address systemic factors that perpetuate discrimination and create an unwelcome space are necessary to reduce stigmatizing attitudes and behaviours. Curriculum on culturally and contextually appropriate care should be integrated into professional training for all healthcare providers and staff from the beginning of their training, in addition to being required periodically as continuing medical education. Further research to understand the perspectives of health care providers in meeting the needs of patients from EDGs is additionally needed, particularly considering the high rates of healthcare provider burnout in the context of the COVID-19 pandemic [40–42]. Finally, participatory research with members of various EDGs is essential to engage in meaningful dialogue about negative ED care experiences, how to prevent them in the future, and how to address them when they do occur.

### Strengths and limitations

Our findings must be interpreted within the context of its limitations. As a single-centre study with a convenience sample of ED users, the results are not generalizable. The study was prone to selection bias because more acutely ill patients, those who were aggressive or threatening towards staff and those who were deemed to not have capacity to give informed consent were not approached for participation, and they may have had different experiences. Further, patients presenting outside of study hours may have had different demographics and experiences of care, and non-English speaking patients were not included. Additionally, participants were limited to choosing up to 3 EGDs when they may have identified with more than three groups. Furthermore, although data were also collected in the community through research partners, some of the most marginalized patients in our community may still have been missed if they were not accessing any care or services. Due to a software update issue, several tablets failed to collect demographic and contextualizing data in the early data collection phase resulting in a substantial, albeit random, proportion of missing data on some multiple-choice questions. Finally, participants were asked about earlier ED experiences which may have introduced recall bias and did not allow us to account for changes in perceptions between the time of the index ED visit and the time of study participation.

The study also has several noteworthy strengths. It is one of the first quantitative studies to examine ED care experiences among members of diverse EDGs and included a comparison group of patients who did not identify as a member of an EDG. Furthermore, members of EDGs were also recruited in the community to be more inclusive of those who may not be seeking care in the ED given earlier negative experiences. Additionally, by nature the sensemaking survey reduces social desirability bias because all possible responses within a given question are either all positive, all negative or all neutral. This reduces the possibility that one response will be viewed as more acceptable than others. A sensemaking approach also reduces interpretation bias because participants are empowered to interpret their own experiences which limits interpretation biases that are inherent when the research team interprets the experience. Finally, the research protocol, including the survey design, used a participatory approach with input from community partners who serve EDGs as well as input from members of the EDGs themselves.

## Conclusion

The current study contributes to the body of literature about how individuals who identify as equity-deserving compared to those who do not experience ED care at a single centre in Ontario, Canada. We identified two areas that can perhaps be more easily addressed to improve care for members of EGDs: (1) improved recognition and attention to patients’ identity, culture, and personal situation (for example by providing ED patients with more opportunity to self-identify their social determinants of health); and (2) treating all patients with kindness and respect. Thematic analysis of the qualitative data collected as part of this mixed-methods study will further help contextualize findings and identify how best to improve ED care experiences among each EDG to be more inclusive and better able to meet their healthcare needs.

## Electronic supplementary material

Below is the link to the electronic supplementary material.

Supplementary Material 1

Supplementary Material 2

Supplementary Material 3

Supplementary Material 4

## Data Availability

The datasets used and/or analysed during the current study are available from the corresponding author on reasonable request.

## Abbreviations

ED: Emergency Department
EDG: Equity-deserving group
KHSC: Kingston Health Sciences Centre
OCAP: Ownership, Control, Access, and Possession
RA: Research assistant
UCC: Urgent care centre
