# Supplementary material for: Emergency department care experiences among members of equity-deserving groups: quantitative results from a cross-sectional mixed methods study
Source: BMC Emerg Med. 2023 Feb 21;23:21. doi: 10.1186/s12873-023-00792-z (PMC9942657; doi:10.1186/s12873-023-00792-z)
Supplement: Supplementary file 1 — Supplementary Material 1 [file 12873_2023_792_MOESM1_ESM.docx]

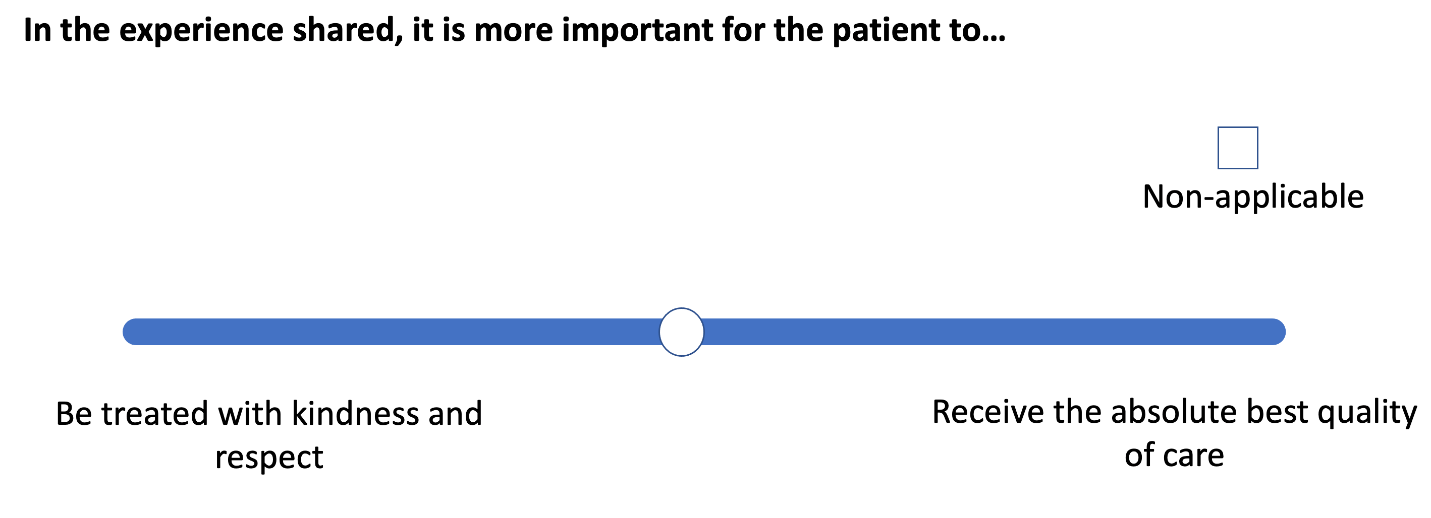


**Example 1: Slider question.**

Participants use their finger to drag the white indicator to plot their perspectives on the ED experience shared in the survey.
