# Supplementary material for: Emergency department care experiences among members of equity-deserving groups: quantitative results from a cross-sectional mixed methods study
Source: BMC Emerg Med. 2023 Feb 21;23:21. doi: 10.1186/s12873-023-00792-z (PMC9942657; doi:10.1186/s12873-023-00792-z)
Supplement: Supplementary file 3 — Supplementary Material 3 [file 12873_2023_792_MOESM3_ESM.docx]

**Appendix 2 - List of Community Partners**

| **Equity-Deserving Population** | **Community Partner** |
| --- | --- |
| Indigenous Peoples | Tyendinaga Interprofessional Primary Care Clinic |
| Indigenous Peoples | Belle Island Indigenous People’s Day Ceremony |
| Persons with disabilities | Ongwanada Resource Centre |
| Persons with disabilities | Kingston Health Sciences Centre’s Vision Rehabilitation Clinic |
| Persons with disabilities | Independent Living Centre Kingston |
| Persons with disabilities + Mental health | Providence Care Hospital - Mental Health & Rehabilitation Departments |
| Mental Health | Addictions and Mental Health Services (AMHS-KFLA) |
| 2SLGBTQ+ | Kingston General Hospital Transgender Clinic |
| Substance use | Change Health Care |
| Substance Use | Brock Street Detoxification Centre |
| Substance use | KCHC - Weller Clinic |
| Substance use | Integrated Care Hub - Consumption and Treatment Services |
| Substance use | Kingston Harbour Light |
| Substance Use | Brock Street Detoxification Centre |
| Substance use | HIV/AIDS Regional Services (HARS) |
| Substance use, Vulnerably housed, and Mental health | Kingston Street Health Centre |
| Vulnerably housed | St. Vincent de Paul Society of Kingston |
| Vulnerably housed | Kingston Youth Shelter |
| Vulnerably housed | One Roof Kingston |
