# Supplementary material for: Emergency department care experiences among members of equity-deserving groups: quantitative results from a cross-sectional mixed methods study
Source: BMC Emerg Med. 2023 Feb 21;23:21. doi: 10.1186/s12873-023-00792-z (PMC9942657; doi:10.1186/s12873-023-00792-z)
Supplement: Supplementary file 4 — Supplementary Material 4 [file 12873_2023_792_MOESM4_ESM.docx]

**Appendix 3. Survey questions with possible responses**

| **Question** | **Possible Responses** |
| --- | --- |
| Micro-narrative prompts | |
| Share an example of how visiting the emergency room helped or harmed you or someone you were at the hospital with. | Micro-narrative recorded by participant |
| Tell a story about the best or worst experience you or someone you were with had in the emergency room. | Micro-narrative recorded by participant |
| Give an example of an experience that went very well or very badly for you or someone you were with at the emergency room. | Micro-narrative recorded by participant |
| Dyads | |
| During the emergency room visit, the patient’s personal situation, identity or culture received… | 1) Far too little attention;  2) Far too much attention  or some combination thereof |
| The events in the story were mostly about… | 1) How the hospital works;  2) Emergency room staff  or some combination thereof |
| Based on the story shared, the patient’s ability to pay for care or other costs (i.e. medicines, travel for care) received... | 1) Far too little attention;  2) Far too much attention  or some combination thereof |
| Based on the story shared, the patient was given... | 1) Too little attention to their needs;  2) Too much attention to their needs  or some combination thereof |
| In the experience shared, it was more important for the patient to… | 1) Receive the best possible medical care;  2) Be treated with kindness and respect  or some combination thereof |
| Triads | |
| During the events in the shared story, the patient was ... | 1) Judged ;  2) Powerless/Not in control;  3) Ignored  or some combination thereof |
| During this story, the patient was... | 1) Informed;  2) Empowered/In control ;  3) Accepted/valued  or some combination thereof |
| In the shared experience, the doctors, nurses or other emergency room staff ... | 1) Understood the situation;  2) Shared important information;  3) Showed they cared  or some combination thereof |
| The patient’s shared experience was affected most by: | 1) Wait times;  2) The medical care/testing provided;  3) How emergency room staff behaved towards the patient  or some combination thereof |
| After the patient left the emergency room, he/she was: | 1) Not sure of what to do next;  2) Unclear about their health condition;  3) Unsupported in coping with their health concern  or some combination thereof |
| Based on the shared story, the following would most improve future emergency room care: | 1) Better understanding of personal situation, identity and culture;  2) Easier access to medical care;  3) Better communication between health care workers  or some combination thereof |
| Star Questions | |
| Pick the stars that relate to your story. Move the stars to a place in the square where they best fit with the patient’s visit. Please read the questions carefully.  Axes:  Y-axis: How important is this to you? (not at all to a lot)  X-axis: How much did the patient experience this? (not at all to a lot) | 1) Follow-up medical care  2) Regular updates  3) Symptoms treated  4) Clear instructions  5) Culturally appropriate care  6) Community resources |
| Pick the stars that relate to your story. Move the stars to a place in the square where they best describe the patient’s visit. Please read the questions carefully.  Axes:  Y-axis: How important is this to you? (not at all to a lot)  X-axis: How much did the patient experience this? (not at all to a lot) | 1) Being respected  2) Feeling comfortable  3) Issue taken seriously  4) Feeling safe  5) Privacy  6) Being understood |
| Multiple Choice Questions About the Shared Experience | |
| Who was the patient in the story (choose only 1)? | 1) It was me  2) It was my child  3) It was my parent / guardian  4) It was my spouse / partner  5) It was someone else in my family  6) It was a friend  7) It was a person for whom I am a caregiver (paid or volunteer)  8) Not sure / prefer not to say  9) Other _________________________ |
| Was the visit in your shared story for concerns related to COVID (choose only 1)? | 1) Yes  2) No  3) Not sure / prefer not to say |
| In what way did COVID change how easy it was for the patient to get care in the shared story (choose only 1)? | 1) It was easier to get the care needed  2) It was harder to get the care needed  3) COVID did not impact the patient’s access to care.  4) Not sure / prefer not to say |
| How did COVID impact the patient’s care experience in the story told (choose only 1)? | 1) The patient’s care experience was better than normal  2) The patient’s care experience was worse than normal  3) COVID did not impact the patient’s care experience  4) Not sure / prefer not to say |
| How did you feel about the patient’s visit overall (choose only 1)? | 1) Very bad  2) Bad  3) Not good or bad  4) Good  5) Very good  6) Not sure / prefer not to say |
| Some groups can face barriers to accessing health care. Which of the following, if any, most relates to the patient in the story (choose up to 3)? | 1) Ethnic minority/ person of colour  2) Indigenous  3) Person with a disability  4) Mental health concern or illness  5) 2SLGBTQ+  6) Alcohol/ drugs or substance use  7) Homelessness/ without stable housing  8) Trading sex for food, money or goods  9) Member of a gang  10) Incarceration (in jail/prison)  11) Sexual assault or experiencing violence  12) None of the above  13) Not sure / prefer not to say  14) Other _______________________ |
| How did the patient’s personal situation, identity, and/or culture affect the experience (choose only 1)? | 1) In a very bad way  2) In a bad way  3) It did not affect the treatment received  4) In a good way  5) In a very good way  6) Not sure / prefer not to say |
| If the patient identifies as Indigenous, to which Indigenous group do they belong (choose only 1): | 1) Patient is not Indigenous  2) First Nations  3) Métis  4) Inuit  5) Other______________ |
| If the patient is a person with a disability, which relates most to the story that the patient shared (choose only 1)? | 1) Patient is not a person with a disability  2) Physical disability  3) Mental health disability  4) Low vision/blindness  5) Hearing loss/deafness  6) Intellectual disability (need help with public transportation, managing money...)  7) Other (learning disability, autism spectrum disorder, neurodiverse...)  8) Not sure / prefer not to say |
| What is the patient’s ethnicity (choose only one)? | 1) Indigenous  2) White /European  3) South Asian (East Indian, Pakistani...)  4) Chinese  5) Black  6) Filipino  7) Latin American  8) Arab  9) Southeast Asian (e.g., Vietnamese, Thai...)  10) West Asian (e.g., Iranian, Afghan...)  Korean  11) Japanese  12) One or more ethnicity  13) Other ________________  14) Not sure/prefer not to say |
| How does the patient identify (choose only 1): | 1) Man  2) Woman  3) Non-binary  4) Not sure / prefer not to say |
| What is the patient’s sexual orientation (choose only 1)? | 1) Straight  2) Gay/lesbian  3) Bisexual  4) Pansexual  5) Asexual  6) Questioning/ Unsure  7) Not sure / prefer not to say  8) Sexual orientation that is not on this list |
| Does the patient identify as gender diverse (transgender, two-spirit, gender fluid, non-binary, or agender) (choose only 1)? | 1) Yes  2) No  3) Not sure/prefer not to say |
| Was the patient in your story (choose only 1): | 1) Less than 18 years of age  2) 18 - 25 years of age  3) 26 - 45 years of age  4) 46 - 65  5) Greater than 65 years of age  6) Not sure / prefer not to say |
| How often does the patient in the story struggle to make ends meet (not enough money for food, bills, housing, clothes...) (choose only 1)? | 1) Never  2) Rarely  3) Sometimes  4) Often  5) All the time  6) Not sure / prefer not to say |
| How long ago did the shared emergency room visit occur? (choose only 1): | 1) 0 – 6 months  2) 7 – 12 months  3) 13 – 18 months  4) 19 -24 months  5) More than 24 months  6) Not sure / prefer not to say |
| How often did the patient in the story go to an emergency room in Kingston in the 24 months prior to the visit shared? (choose only 1): | 1) Did not access care in the emergency room before the experience described  2) 1 – 3 times  3) 4 – 6 times  4) 7 – 9 times  5) 10 or more times  6) Not sure / prefer not to say |
| The events in the story were mostly focused on what part of the emergency room (choose only 1): | 1) Triage (where nurse took your blood pressure)  2) Registration (where a clerk registered your health card)  3) Waiting room  4) Nursing staff  5) Doctors  6) Social workers  7) Porters, x-ray and/or CAT scan technicians  8) Security officers  9) Discharge  10) Not sure / prefer not to say  11) Other ________________________ |
| How often do you think the situation in your story occurs (choose only 1): | 1) It is very rare  2) It happens from time to time  3) It is somewhat typical  4) It happens all the time  5) Not sure / prefer not to say |
| How did your story make you feel at the time it occurred (choose up to 3): | 1) Accepted  2) Afraid  3) Disappointed  4) Embarrassed  5) Frustrated  6) Happy  7) Helpless  8) Hopeful  9) Relieved  10) Satisfied  11) Thankful  12) Worried  13) Not sure / prefer not to say |
| Is the story about the patient being treated without respect or the patient being judged (choose only 1)? | 1) Yes  2) No  3) Not sure / prefer not to say |
| Multiple Choice Questions About the Research Participant | |
| What is your age (choose only 1): | 1) 16 – 24  2) 25 - 34  3) 35 – 44  4) 45 – 54  5) 55 - 64  6) 65 or older  7) Not sure / prefer not to say |
| What is your ethnicity? | 1) Indigenous  2) White /European  3) South Asian (East Indian, Pakistani...)  4) Chinese  5) Black  6) Filipino  7) Latin American  8) Arab  9) Southeast Asian (e.g., Vietnamese, Thai...)  10) West Asian (e.g., Iranian, Afghan...)  Korean  11) Japanese  12) One or more ethnicity  13) Other ________________  14) Not sure/prefer not to say |
| How do you identify (choose only 1)? | 1) Man  2) Woman  3) Non-binary  4) Not sure / prefer not to say |
| Where did you learn about this study (choose only 1): | 1) Kingston General Hospital  Emergency Department  2) Hotel Dieu Hospital Urgent Care Centre  3) Word of mouth  4) Shared online through social media  5) Other organization |
| Comments or anything else you would like to share about your emergency room visit | Free text field |
| *Response was optional for all questions. | |
